# Understanding client and provider perspectives of antenatal care service quality: a qualitative multi-method study from Tanzania

**DOI:** 10.7189/jogh.09.01101

**Published:** 2019-06

**Authors:** Ashley Sheffel, Rebecca Heidkamp, Rose Mpembeni, Peter Bujari, Jaya Gupta, Debora Niyeha, Tricia Aung, Victor Bakengesa, John Msuya, Melinda Munos, Caitlin Kennedy

**Affiliations:** 1 Johns Hopkins Bloomberg School of Public Health. Baltimore, Maryland, USA; 2 Muhimbili University of Health and Allied Sciences, Dar es Salaam, United Republic of Tanzania; 3 Health Promotion Tanzania. Dar es Salaam, United Republic of Tanzania; 4 Ministry of Health, Community Development, Gender, Elderly, and Children, Dar es Salaam, United Republic of Tanzania; 5 Sokoine University of Agriculture, Morogoro, United Republic of Tanzania

## Abstract

**Background:**

Measures of quality of care in low- and middle-income countries (LMICs) rarely include experience of care. This gap in service quality metrics may be driven by a lack of understanding of client and provider perspectives. Understanding these perspectives is a critical first step in not only improving metrics, but also in improving service delivery. This study identifies the items antenatal care (ANC) clients and health care providers in Tanzania associate with a quality ANC service and explores the experience of care domain from both client and provider perspectives.

**Methods:**

We conducted semi-structured interviews with15 providers and 35 clients in Tanzania that included a free-listing activity to elicit items clients and providers associate with quality ANC services. We analyzed the free-listing for rank order and frequency to identify the most salient items, which were included in the second phase of data collection. We then conducted semi-structured interviews with a pile sort activity with the same 15 providers and 32 new clients to understand the importance of the items identified in the free-listing. We used a thematic analysis driven by the framework approach to analyze interview data.

**Results:**

Both clients and providers perceived quality of ANC as being comprised of items related to experience of care, provision of care, and cross-cutting essential physical and human resources. The free-listing findings illuminated that the experience of care was equally important to clients and providers as the availability of physical and human resources and the content of the care delivered. In addition, clients and providers perceived that a positive patient care experience – marked by good communication, active listening, keeping confidentiality, and being spoken to politely – increased utilization of health services and improved health outcomes.

**Conclusions:**

The experience of care in LMICs is an overlooked, yet critically important topic. Understanding the experience of care from those who receive and deliver services is key to measuring and improving the quality of ANC. Our research highlights the importance of incorporating experience of care into future quality improvement activities and quality measures. By doing so, we identify barriers and facilitating factors of practical use to policy-makers and governments in LMICs.

Reducing maternal mortality has been a long-standing global priority. Monitoring progress towards reductions in mortality has often focused on measuring service coverage defined as the proportion of the population at risk that receives an intervention [1]. Although coverage measures are widely used in maternal health, these measures often focus on service contacts as opposed to the interventions delivered during those contacts. Additionally, in many countries, high maternal mortality persists despite considerable improvements in the coverage of maternal health services. This disconnect between disease burden and intervention coverage underscores the critical role that quality of care plays in improving health outcomes [2-4].

As the international community strives to achieve global reductions in maternal mortality, accurate assessment of quality of care is essential. The World Health Organization (WHO) expert working group for maternal and newborn health has defined quality of care as, “the extent to which health care services provided to individuals and patient populations improve desired health outcomes. In order to achieve this, health care must be safe, effective, timely, efficient, equitable and people-centered” [5-7]. The group also devised a conceptual framework for quality of care, the WHO Quality of Care Framework for Maternal and Newborn Health (WHO framework) [7]. A novel feature of the WHO framework is that it gives equal focus to both provision of care and experience of care, which highlights a global shift toward health systems that are patient-centered in addition to being technically sound. However, the working group did not develop standardized indicators for measuring quality of care across the continuum of care for mothers.

Experience of care is important to the provision of a quality health service, as patient experiences both have intrinsic value and also are an important driver of health outcomes [8]. Patients who have a positive care experience are more likely to receive accurate diagnoses and treatment, increase their adherence to provider recommendations and treatment, and continue to utilize health services [9]. While formative qualitative research to understand patient perceptions of service quality has been carried out in developed settings, there has been little research in this area in developing countries [8,10]. Research conducted in low- and middle-income countries (LMICs) has focused on creating valid, reliable instruments to quantitatively measure perceived quality of care [11,12]. However, these studies often do not take into account patient and provider perspectives, making it is difficult to know if perceived quality of care is being measured accurately. While there are tools and indicators being used in LMICs to measure quality of care, such as the Service Provision Assessment (SPA) and the Service Availability and Readiness Assessment (SARA), these tools often focus on assessing the availability of facility inputs such as physical and human resources and the provision of care as compared to best practices based on clinical guidelines. Rarely do these tools include assessment of the client’s experience of care. There is a growing body of literature on defining and measuring the client experience during intrapartum care in developing countries [13-15]. However, there is little evidence on how to measure experience of care across the continuum of maternal care which is particularly important for antenatal care (ANC) given that it may encourage or discourage women from delivering at health facilities. The absence of care experience in existing service quality metrics may be driven by both a lack of data as well as by a lack of understanding of client and provider perspectives of quality of care. Understanding these perspectives is a critical first step in not only improving quality of care metrics, but also improving service delivery.

We address the gap in the quality of care literature by gathering data in Tanzania on client and provider perspectives of ANC service quality. As part of an evaluation of the National Road Map Strategic Plan To Accelerate Reduction of Maternal, Newborn and Child Deaths in Tanzania ("One Plan"), the government strategy to accelerate reduction of maternal, newborn, and child deaths from 2008 to 2015, the National Evaluation Platform (NEP) project recognized the need to comprehensively measure the quality of ANC. As part of the NEP project, this study aimed to identify the items clients and providers associate with a quality ANC service and explore the experience of care domain from both client and provider perspectives.

## METHODS

### Study site and participant selection

We selected study sites using data from the Tanzania Service Provision Assessment (TSPA) and the Health Management Information System (HMIS) to identify a region with both high-quality and low-quality ANC services. The facilities were stratified by level of care to maximize variation in respondents’ perceptions and beliefs about the quality of ANC services, which may vary by health facility quality. We restricted the sample to public primary health facilities (health centers and dispensaries) located in mainland Tanzania that offer ANC services. For this subset of facilities, we calculated an ANC quality of care score based on the mean availability of eleven items assessed in the 2015 TSPA. We ranked facilities according to their scores, then classified the top and bottom 15% of health centers and dispensaries as “high-quality facilities” or “low-quality facilities,” respectively. Using this scoring method, we examined each of the twenty-five regions in mainland Tanzania to identify those with at least one facility in each of four categories: high-quality health center, high-quality dispensary, low-quality health center, low-quality dispensary. From these eligible regions, we excluded regions that had distinct ethnic or cultural composition and unique socioeconomic characteristics in order to improve transferability. In addition, regions that were not feasible due to remoteness were excluded. Next, we examined the health facilities in each region and identified regions with at least two health centers and two dispensaries for which there was facility-level data on ANC service volume available in the HMIS and the total number of first ANC visits reported by the facility was at least 75 in 2015. Based on these criteria, we selected Tanga, a rural northeast region. We then purposively selected one high-quality health center, one high-quality dispensary, one low-quality health center, and one low-quality dispensary located within Tanga. Characteristics of selected health facilities selected are found in **Table 1****.** All health care providers delivering ANC services, present on the day of the interview, and who were at least 18 years or age were eligible for to participate in the study.

**Table 1:** Health facility demographic information

Demographic features	Facility 1: High-quality Dispensary	Facility 2: Low-quality Dispensary	Facility 3: High-quality Health Center	Facility 4: Low-quality Health Center
District	Muheza DC	Muheza DC	Mkinga DC	Muheza DC
Facility type	Dispensary	Dispensary	Health Center	Health Center
Urban/rural	Rural	Rural	Rural	Rural
Population catchment area (2016)	5678	3267	3604	6512
ANC 1 Volume (2015)	75	102	212	233
ANC 4 Volume (2015)	23	23	23	84
ANC Quality Score	0.64	0.27	0.82	0.46

ANC – antenatal care, DC – district council

We used convenience sampling to select two hamlets in the catchment area of each health facility. Within these hamlets, women 18 years of age or older, who had experienced a birth in the last two years and received ANC services at the identified facilities, were eligible to participate. We used purposive sampling during each phase to recruit women with less than four ANC visits and women with four or more ANC visits during their last pregnancy. In determining sample size, we followed guidelines which suggested 20 to 30 free-list informants and 30 to 40 pile sort informants would be sufficient to reach saturation [16]. The FLAME software data saturation analysis feature was also used to assess when additional participants contributed few or no new items to the free-listing [17]. Data collection for both phases ended when saturation was reached and little to no new information was gained from additional interviews.

### Data collection and analysis

All interviews were conducted in Kiswahili by the local research firm Health Promotion Tanzania – formerly known as Human Development Trust and currently known by the acronym HDT. All team members had prior qualitative research experience and were trained in the study methods. English and Kiswahili versions of the interview guides were reviewed after pre-testing to ensure questions were accurately translated, understandable, and followed a logical flow.

In the first phase, researchers conducted semi-structured interviews employing an embedded free-listing component with clients and providers, each lasting 30-40 minutes. Free-listing is a technique used to gather emic perspectives in which a respondent is asked about a domain and responds with a ‘list’ that represents elements in that domain [16,18]. Free-listing was used to elicit items clients and providers associated with a quality ANC service. Participants were asked, “Can you list all the elements that you associate with a good quality ANC service?” Once the participant finished listing all items, the interviewer asked for a brief explanation of each item on the list.

Analysis of the free-list data was performed using FLAME software, a Microsoft Excel add-on designed specifically for the analysis of free list data [17,19]. Each respondent’s list was entered into the Excel workbook. The research team examined the data to standardize language and to group terms with similar meanings across participant lists. For each item, analysis was conducted for both rank order and frequency to identify the most salient items [20]. The Smith index, a measure of salience ranging from zero representing low salience to one representing high salience, was calculated [21,22]. In addition, a scree plot was generated which shows a natural break between the highly salient items and the items mentioned by only person [23]. We identified the natural break of the scree plot to aid in identification of the most salient items. The analysis was implemented for clients, providers, and clients and providers combined. Items with the highest salience and those of particular interest to researchers were included in the second phase of data collection.

In the second phase of data collection, researchers conducted semi-structured interviews with an embedded pile sorting component with providers and clients, each lasting 60-90 minutes. The pile sorting component was conducted to gain an understanding of the relative importance of the components of a quality ANC service for clients and providers. Pile sorting is a methodology used to understand people’s perception of a cultural domain through observation of how people group the items of the domain of interest [24]. The structured pile sort required participants to categorize components of a quality ANC service into four groups based on importance. These items were identified through the free-listing as being the most salient items within the domain of “quality ANC service components.” The pile sort required the participant to consider each item, which were written on cards in Kiswahili, and separate them into piles of important and non-important items. Within each pile, the participant was again asked to sort items into two groups based on relative importance. At the end of the exercise, each participant had a total of four piles ranked by importance. After sorting, the interviewer probed to understand why each item was perceived to be important.

Audio-recorded interviews were transcribed and translated from Kiswahili into English by the HDT research team. To assess quality of transcription and translation, 10 interviews were randomly selected for data quality checking. All discrepancies were minimal and resolved prior to analysis. Data management, coding, and thematic analysis were performed using the web-based software Dedoose v8.0 (www.dedoose.com) and Microsoft Excel (Microsoft Inc, Seattle, WA, USA) [25]. A thematic analysis driven by the framework approach was used to analyze interview data from the second phase of data collection [26]. First, the two-member research team familiarized themselves with the interviews by reading and open-coding four transcripts each (2 providers and 2 clients). Based on open coding of initial transcripts, the research team developed an analytical framework with seven themes. The analytical framework was applied to each transcript with the research team meeting weekly to discuss coding challenges or questions that emerged. The same process of open coding, development of an analytical framework, and applying the analytical framework was undertaken for the sub-codes within each theme. Once all data was coded, it was charted into a framework matrix in Microsoft Excel. The framework matrix was used to synthesize and interpret the results. After analysis, we organized the results and themes according to an existing globally-recognized conceptual framework for quality of care – the WHO Framework [5,7]. Finally, a local workshop with individuals from the Ministry of Health, National Bureau of Statistics, and local research institute partners was held as a form of member checking.

### Ethical considerations

The study was approved for ethical clearance by both the Institutional Review Boards of the Johns Hopkins School of Public Health in Baltimore, Maryland (IRB No. 00007487) and the National Institute for Medical Research in Dar es Salaam, Tanzania (Ref. No. NIMR/HQ/R.8a/Vol. IX/2378). All potential study participants underwent a verbal voluntary informed consent process.

## RESULTS

The characteristics of the study participants are in **Table 2**. In phase one, interviews were conducted with 15 providers and 35 clients. In phase two, interviews were conducted with the same 15 providers as in phase one and 32 new clients. Services in primary health facilities in Tanzania are supposed to be delivered by lower cadre clinical staff such as clinical officers and nurses, since medical doctors are rarely posted to primary care facilities. However, due to the shortage of human resources for health in Tanzania, there are also non-clinical staff such as medical attendants who deliver services even though they lack professional training. In our study, two-thirds of the providers interviewed were clinical staff (one clinical officer, nine nurses), while one-third of providers interviewed were non-clinical staff (five medical attendants). Women with a range of ANC experiences were interviewed with approximately a quarter of women in each of the four categories (4+ ANC visits, High-quality facility; <4 ANC visits, High-quality facility; 4+ ANC visits, Low-quality facility; <4 ANC visits, Low-quality facility).

**Table 2:** Characteristics of study participants

Participant characteristics	Providers (n = 15)	Clients phase 1 (n = 35)	Clients phase 2 (n = 32)
**Average age** (years)	38 (range: 26-53)	28 (range: 18-43)	29 (range: 18-46)
**Gender:**			
Female	13 (87%)	35 (100%)	32 (100%)
Male	2 (13%)		
**Participant group:**			
Woman- 4+ ANC visits, High-quality facility		8 (23%)	7 (22%)
Woman- <4 ANC visits, High-quality facility		10 (29%)	7 (22%)
Woman- 4+ ANC visits, Low-quality facility		9 (26%)	9 (28%)
Woman- <4 ANC visits, Low-quality facility		8 (23%)	9 (28%)
Provider- High-quality Health Center	5 (33%)		
Provider- Low-quality Health Center	5 (33%)		
Provider- High-quality Dispensary	2 (13%)		
Provider- Low-quality Dispensary	3 (20%)		
**Level of education:**			
No education		6 (17%)	1 (3%)
Some primary school		3 (9%)	2 (6%)
Complete primary school		20 (57%)	21 (66%)
Some secondary school		1 (3%)	3 (9%)
Complete secondary school		5 (14%)	5 (16%)
**Average number of ANC visits**		3.7 (Range: 2-6)	3.5 (Range: 1-6)
**Average number of children**		2.8 (Range: 1-6)	1.7 (Range: 1-8)
**Professional qualification:**			
Ward/Medical attendant	5 (33%)		
Nursing assistant/attendant	2 (13%)		
Enrolled nurse	3 (20%)		
Registered nurse	4 (27%)		
Clinical officer	1 (7%)		

ANC – antenatal care

### Items associated with a quality ANC service

The free-listing activity identified 25 items each for clients and providers that were considered to be important for a quality ANC service. A total of 36 unique items were listed between the two groups, with overlap occurring for 14 of these items. Client free-lists averaged five items while provider free-lists averaged seven items. The items, frequency, average rank, and salience for client and provider free-listing can be found in **Table 3** and **Table 4**, respectively.

**Table 3:** Item, frequency, average rank, and salience for ANC client free-listing

Item	Frequency*	Average rank†	Salience score‡
Health providers treat clients respectfully such as not shouting and not being rude	70.6%	3.00	0.47
Facility provides medicines such as SP, ferrous, folic acid, mebendazole, TT vaccine, ARVs, and antibiotics	70.6%	3.13	0.43
Health provider delivers a complete physical examination such as height and weight measurement, fetal heart rate, blood pressure, abdominal examination	64.7%	2.96	0.44
Facility provides laboratory tests such as hemoglobin, malaria, urine protein, blood group, HIV, and syphilis	50.0%	2.71	0.34
Health care workers provide women with health education on topics such as family planning, pregnancy, nutrition, and birth preparedness	35.3%	2.83	0.24
Facility has adequate trained staff for example: RCH, ANC, laboratory, ultrasound technician	23.5%	2.88	0.15
Health facility has supplies such as gloves, mackintosh, cotton, and cord ties	26.5%	4.22	0.13
Health providers listen to the client such as being attentive to their complaints	20.6%	3.71	0.12
Services provided for free to pregnant women	11.8%	2.25	0.08
Facility has adequate equipment such as blood pressure machine, weighing scale, thermometer, fetal scope, examination bed, tape measure, and ultra sound machine	17.6%	3.67	0.10
Facility attends to pregnant women in a timely manner	20.6%	4.71	0.09
Facility provides client amenities such as clean service area, beds with clean sheets and nets, toilets, chairs for waiting, water, ventilation	17.6%	5.17	0.06
There is good communication between service provider and client	11.8%	3.50	0.07
Facility has adequate infrastructure such as enough service and waiting rooms	11.8%	5.50	0.04
Providers issue an ANC card and record all services delivered at each visit	5.9%	3.50	0.04
Facilities have transport for referral such as an ambulance	2.9%	2.00	0.03
Providers allocate sufficient time to each client	2.9%	2.00	0.03
Women have health insurance	5.9%	5.00	0.03
Facility provides accurate testing results	2.9%	3.00	0.02
Facility distributes ITNs or vouchers to pregnant women	2.9%	3.00	0.01
Nurses should be motivated to provide better care to women	2.9%	4.00	0.02
Nurses are present at delivery	2.9%	6.00	0.01
Providers should give priority to those who are the sickest	2.9%	6.00	0.01
Women should attend all ANC appointments	2.9%	6.00	0.01
Health providers attend to women who arrive with or without a partner	2.9%	8.00	0.00

SP – sulphadoxine-pyrimethamine, TT – tetanus toxoid, ARV – antiretroviral, HIV – human immunodeficiency virus, RCH – reproductive and child health, ANC – antenatal care, ITN – insecticide treated net

*Frequency is the proportion of participants who listed the item

†Average rank is the position in which a word appears in a list, averaged across all participants

‡Salience was derived using Smith’s S which accounts for both the frequency and the order of the words

**Table 4:** Item, frequency, average rank, and salience for health care provider free-listing

Item	Frequency*		Average rank†	Salience score ‡
Facility has adequate trained staff for example: RCH, ANC, laboratory, ultrasound technician	86.7%	2.23		0.72
Facility has adequate equipment such as blood pressure machine, weighing scale, thermometer, fetal scope, examination bed, tape measure, and ultra sound machine	73.3%	3.55		0.45
Facility provides medicines such as SP, ferrous, folic acid, mebendazole, TT vaccine, ARVs, and antibiotics	66.7%	4.20		0.38
Facility has adequate infrastructure such as enough service and waiting rooms	66.7%	4.00		0.38
Facility provides laboratory tests such as hemoglobin, malaria, urine protein, blood group, HIV, and syphilis	53.3%	3.75		0.31
Health care workers provide women with health education on topics such as family planning, pregnancy, nutrition, and birth preparedness	46.7%	5.00		0.24
Service providers receive in-service training to keep skills current	40.0%	4.83		0.21
There is good communication between service provider and client	40.0%	4.33		0.24
Facilities have transport for referral such as an ambulance	26.7%	5.25		0.12
Health provider delivers a complete physical examination such as height and weight measurement, fetal heart rate, blood pressure, abdominal examination	20.0%	5.67		0.10
There is a good working environment for health care providers for example staff housing, cleanliness, spacious and ventilated rooms	20.0%	5.33		0.11
Staff are compensated for working overtime	20.0%	5.67		0.09
Health providers treat clients respectfully such as not shouting and not being rude	20.0%	5.00		0.13
Facility attends to pregnant women in a timely manner	13.3%	4.00		0.09
Facility properly records data on services provided to women on ANC card and registers	13.3%	6.50		0.04
Providers keep patient confidentiality	13.3%	4.00		0.08
Health facility provides outreach services to pregnant women	13.3%	5.00		0.04
Collaboration among facility staff to share workload	13.3%	3.00		0.09
Health providers encourage couples to attend ANC together	13.3%	5.50		0.04
Health facility has supplies such as gloves, mackintosh, cotton, and cord ties	13.3%	4.50		0.07
Facility provides client amenities such as clean water	13.3%	6.00		0.05
Health providers and community have shared goal for encouraging ANC	6.7%	6.00		0.05
Waiting home is available at the facility	6.7%	6.00		0.03
Health providers listen to the client such as being attentive to their complaints	6.7%	8.00		0.02
Providers allocate sufficient time to each client	6.7%	4.00		0.04

SP – sulphadoxine-pyrimethamine, TT – tetanus toxoid, ARV – antiretroviral, HIV – human immunodeficiency virus, RCH – reproductive and child health, ANC – antenatal care, ITN – insecticide treated net

*Frequency is the proportion of participants who listed the item.

†Average rank is the position in which a word appears in a list, averaged across all participants.

‡Salience was derived using Smith’s S which accounts for both the frequency and the order of the words.

When asked what elements are important for an ANC service to be of good quality, both clients and providers listed items related to provision of care (complete physical examination, health education, and transportation for referral), experience of care (good communication, listening to the client, treating clients respectfully, attending to clients in a timely manner, and allocating sufficient time to each client), and availability of essential physical and human resources (medicines, laboratory capacity, equipment, infrastructure, supplies, and trained staff). While there was overlap in the items listed by clients and providers, the ranking of items based on salience score differed between groups. For example, the most salient item for clients was “health care providers treat clients respectfully” while the most salient item for providers was “facility has adequate trained staff.” Both clients and providers listed items that were uniquely salient to their respective group. For example, clients listed “facility provides client amenities” and providers listed “good working environment for health care providers.” Twenty-two items were selected for use in the second phase of data collection. The selected items are presented in **Table 5**, organized according to the WHO Framework. Of the 22 items, 14 items were listed by both clients and providers, three items by clients only, and five items by providers only.

**Table 5:** Free-listing items selected

Framework dimension		Framework domain	Items associated with a quality antenatal care service	Client	Provider
**PROVISION OF CARE**		Evidence-based practices for routine care and management of complications	Health care worker delivers a complete physical examination	X	X
			Health care workers provide women with health education	X	X
		Actionable information systems	Facility properly records data on services provided to women		X
		Functional referral systems	Facilities have transport for referral such as an ambulance	X	X
**CROSS-CUTTING: ESSENTIAL PHYSICAL RESOURCES AVAILABLE**			Facility provides medicines	X	X
			Facility provides laboratory tests	X	X
			Facility has adequate equipment	X	X
			Facility has adequate infrastructure	X	X
			Health facility has supplies	X	X
			Services provided for free to pregnant women	X	
			Facility provides client amenities	X	
**CROSS-CUTTING: COMPETENT, MOTIVATED HUMAN RESOURCES**			Facility has adequate trained staff	X	X
			Health care workers receive in-service training		X
			Good working environment for health care workers		X
**EXPERIENCE OF CARE**		Effective communication	Good communication between health care worker and client	X	X
			Health care workers listen to the client	X	X
		Respect and preservation of dignity	Health care workers treat clients respectfully	X	X
			Health care workers keep patient confidentiality		X
			Facility attends to pregnant women in a timely manner	X	X
			Health care workers allocate sufficient time to each client	X	X
		Emotional support	Health care workers attend to women who arrive with or without a partner	X	
			Health care workers encourage couples to attend ANC together		X

### Client and provider perspectives on experience of care in Tanzania

Clients and providers shared their perspectives on experience of care in Tanzania, including which components of a quality ANC service were most important. Both clients and providers had difficulty ranking items as they thought most of the items were important. We did not find notable differences between high-quality and low-quality facilities, nor between women who received less than four ANC visits compared to those who received four or more ANC visits. As such, we present the results only disaggregating between clients and providers.

The reasons clients and providers gave for an item being an important component of a quality service were grouped into seven themes listed in **Table 6**. However, focusing on the experience of care domain items, four main themes emerged: non-discrimination, access to care, client experience/satisfaction, and client-provider relationship. While the analysis was conducted by examining themes, we present the results below by item and organized according to the WHO Framework experience of care domains: effective communication, respect and preservation of dignity, and emotional support.

**Table 6:** Main themes emerging from qualitative analysis

Theme	The reason I think it is an important component of a quality service is because...
Expectations	“…it is my expectation”
Non-discrimination	“…clients should not be discriminated against”
Access to care	“…all people should have access to care”
Client experience/satisfaction	“…clients should feel comfortable and satisfied”
Provider resources	“…it enables me to do my job well”
Provider motivation	“…it motivates me in my job”
Client-provider relationship	“…it builds a relationship with my provider/client”
Health outcomes	“…it will ensure my/my baby's health during pregnancy”

#### Effective communication

##### Free-list item: Good communication

Clients and providers highlighted the importance of good communication to a high-quality ANC service. Clients associated good communication with trust in the provider. Clients reported that if a provider failed to communicate well they felt uncomfortable with prescribed treatments.

“If proper instructions are not provided to a client, one may take four tablets at once instead of two tablets twice a day. It can lead to another disorder or affect the baby in the womb.” (Client, <4 ANC visits, Low-quality dispensary)

Similarly, providers felt that good communication with clients builds rapport and trust. Providers also recognized that good communication helped them to understand clients’ problems so treatment could be individualized. Finally, providers acknowledged the importance of good communication to ensure the health of the pregnant woman and baby. For example, providers stated that it is important to clearly communicate information such as danger signs during pregnancy so the client can understand how to protect herself and the baby.

“If there is good communication when you give her results she will be satisfied and also if you give her medicine, she will use them properly. If there is not good communication she will have doubts. For example, in providing results on sexual transmitted diseases, others do not know their status, so if you fail to communicate well, a patient may assume that you have forged the results, but if there was a good communication from the start there would be no problem in providing results and if you recommend her to use some medicines she will use [them].” Provider, Low-quality health center)

Both clients and providers indicated that good communication facilitated continued utilization of ANC services.

“When there is a good communication between pregnant mother and service providers, it will motivate a pregnant mother to explain in detail their problem and be motivated to come back to attend clinic. But at times you find a provider is rude and pregnant women may decide not to attend because of that.” (Client, 4+ ANC visits, Low-quality dispensary)

##### Free-list item: Listening attentively

Both clients and providers recognized that it is important for providers to listen to clients. Clients felt the providers must listen actively as it instills confidence that the treatment is tailored to the client’s health needs. In addition, clients expressed that when providers listen, they feel encouraged to share information more openly. Clients stated that trust built through active listening prevented feelings of humiliation and discrimination when sharing sensitive personal information. However, clients noted that while having a provider listen attentively is important, it often does not happen in practice. As a result, some clients felt attentive listening was only somewhat important in relation to service delivery which was considered most important.

“[Attentive listening] is very important because, once the pregnant woman feels privileged at [the] clinic, she gets encouraged, she doesn’t feel discriminated or humiliated. They will not be worried as they feel prioritized. Sometimes the pregnant mother has their own ideas and they may want to ask questions to a nurse. It’s better for a nurse to listen carefully and answer it well.” (Client, <4 ANC visits, Low-quality dispensary)

Providers described that listening to clients and giving clients time to speak allowed them to better understand a client’s medical needs. In addition, they noted that pregnant women are an at-risk population and thus it is important to listen carefully to identify potential complications. Providers also noticed that when clients are listened to, they are more satisfied with the services they receive.

“[Listening attentively] is very important because you are supposed to give her a chance to explain herself. If she has a problem and you are speaking to her so quickly she will not be able to tell you her problems very correctly. So, you should give her enough time to explain herself correctly that she has a certain problem. Then you determine how you should help her. If you go with her very quickly she will leave with her problems unspoken just because you were rushing her.” (Provider, Low-quality dispensary)

Clients and providers alike discussed the importance of listening attentively to improve health outcomes. They stated that by listening to pregnant women, providers were more likely to identify complications and reduce the risk of miscarriage, maternal mortality, and neonatal mortality.

“If you’re not carefully [listening] for example you can find a mother has a problem and you didn’t listen to her well, that mother cannot get a solution to her problem so later when she come to deliver she might have trouble and sometime lose her baby and later can even herself die.” (Provider, Low-quality health center)

#### Respect and preservation of dignity

##### Free-list item: Maintaining confidentiality

Clients and providers emphasized the importance of confidentiality to providing high-quality services. Both groups noted that breaking confidentiality was against the law. Providers further recognized that keeping confidentiality was part of the oath of health care professionals and felt a personal responsibility to honor that commitment. In addition, both groups stated that if a provider or facility violated this confidentiality pact, women would not seek further services.

“It should be a secret between a nurse and woman. It is forbidden to expose [their secrets].” (Client, 4+ ANC visits, High-quality dispensary)

“It is very important to keep patients’ secrets. If you don’t, you will lose a mother from attending clinic and she will fail to make a follow-up of her health and the baby’s progress. She won’t attend clinic, she won’t get required tests, she doesn’t know the progress of baby’s health, and she doesn’t know the progress of her health. That means she will miss health education, she will miss all services because she knows in the facility there is no confidentiality.” (Provider, Low-quality health center)

While clients felt confidentiality was very important, several clients reported that providers do not always keep confidentiality. This was a reason why some clients felt confidentiality was only somewhat important in relation to service delivery which was considered most important.

There is no confidentiality. Because there is no secrecy. If there was, we would not be hearing things in the streets such as that pregnant woman is sick. If she didn’t tell people, how did they know? Do you think a patient can say to people that “I went to check and I was detected HIV positive”? I go to test and detected HIV positive, then it will only be my secret and my husband’s secret. But people point fingers, “that one is sick, she got tested and she is affected.” So, it must be them [providers] telling people. It’s the service providers who expose these secrets. (Client, <4 ANC visits, Low-quality dispensary)

##### Free-list item: Using polite language

Clients and providers emphasized the importance of providers using polite language with clients. Clients stated that when they were spoken to with polite language they were more satisfied with the service and were motivated to return for follow-up services.

“If a nurse has sympathy and love to a pregnant mother, it encourages her. She gets hope even if she may have big problems but as long as she was cared well with love and respect then she somehow feels okay.” (Client, <4 ANC visits, Low-quality health center)

Similarly, providers recognized that if they treat clients with respect and use polite language, a client would be open to share problems and return for follow-up services. In addition, providers noted that if they used impolite language, the facility would gain a bad reputation within the community and pregnant women would not return to seek services.

“If you don’t have love, patients will refuse your services. If you shout to her she will never come back to you. But if you attended her with love and care every time, she will come to you.” (Provider, High-quality dispensary)

Clients said that while having a provider use polite language is important, it often does not happen in practice. This was a reason why some clients felt using polite language was only somewhat important in relation to service delivery which was considered most important.

“Providers are told not to be harsh, they should attend us with love. Thus, why even if you are with your partner, he has to comfort you and not tell you harsh words, but when you reach there they yell at you…We are used to our environment. We don’t know another environment. Because we are sick and we have to go like that even though they yell at us with their unfamiliar language. They tell us that we like to have a lot of children; it is like they abuse you.” (Client, <4 ANC visits, High-quality health center)

##### Free-list item: Non-discrimination

Both clients and providers said that all clients should be treated equally. Providers reported that it is not acceptable to discriminate or deny services based on socioeconomic status. However, some clients reported that those with a lower socioeconomic status receive a lower quality service and are treated disrespectfully. In addition, socioeconomic status was a barrier to services for clients who could not purchase required supplies. This lack of preparation sometimes resulted in denial of services, especially at the time of delivery.

“[It] is most important for pregnant women to get all necessary services. Sometimes a pregnant woman can come and doesn’t have money to get services, so I see it is better for the service to be provided freely for pregnant women because she will get everything. And if she has a problem it will be discovered and there will be no discrimination between those who have and who doesn’t have.” (Provider, Low-quality health center)

##### Free-list item: Timely provision of services

Clients and providers both identified the importance of attending clients in a timely manner. Long wait times were identified as a cause for discomfort for pregnant women and were disruptive to their schedules.

“[Providing timely services] is most important because when pregnant mothers come to clinic they must be provided with services quickly for the purpose of returning home in a timely manner. Some mothers come from far and when they delay to receive services it will cost them to return home.” (Client, <4 ANC visits, Low-quality dispensary)

Providers highlighted limitations when there were few providers and many clients. Some providers felt that it was less important to provide services in a timely manner because it was more important to give each client sufficient time than to ensure clients were not waiting.

“It is not wise to serve the client in a hurry so that others can also have the service. It is advised to have enough time with the mother so that you get to understand her problems and explanations from her. So, for some you might delay. So, it mostly depends on the magnitude of the mothers and their problems, also the number of the care providers. In places where there are few, the process can take a longer time.” (Provider, Low-quality health center)

##### Free-list item: Duration of service

Clients and providers held mixed views on the importance of allocating sufficient time for each client. One shared viewpoint was that it was important to allocate sufficient time for an ANC visit. In this manner, all services could be delivered and the woman could explain her problems and receive care based on individual need.

“Every person has her own problem, it is important to listen to her. We need to have enough time. We are told that you need to be with pregnant woman for the time more than a half an hour. So, it depends. Others have lots to discuss; others have little to discuss. In short you need to listen to her from the start to the end.” (Provider, High-quality health center)

Another shared view was that all clients deserve to be served. Due to the shortage of providers, if the provider spent too much time with one client, others would not receive services. As such, there was a preference for shorter visits for all clients over allocating more time to a select number of clients.

“Let’s say every pregnant mother has their own time, maybe one hour per mother, and you have almost twenty pregnant mothers, how do they receive the services? It means some pregnant women will not receive the service at all.” (Client, 4+ ANC visits, Low-quality dispensary)

“We serve many clients compared to available providers. This is a national crisis. So you can’t spend one hour with one pregnant woman while you have hundreds of pregnant women waiting for you outside. What is important is to provide all services effectively very fast.” (Provider, Low-quality health center)

#### Emotional support

##### Free-list item: Partner presence

The Tanzania national ANC guidelines acknowledge the important role of male participation in reproductive health services. In recent years, partner presence was promoted at ANC visits by government officials. This messaging created confusion that bringing a male partner to the visit was a mandatory precondition to receive ANC services. While some clients recognized that they had a right to receive services regardless, others believed that partner presence was compulsory. Clients felt that all pregnant women should receive services regardless of partner presence since partners may be traveling or away for work or because not all pregnant women have a partner. Consequently, the absence of a partner should not infringe on a pregnant woman’s autonomy and ability to access health services.

“They should serve us as we are. If you don’t go with your partner, currently you will be rejected in our hospital. The health providers, you apologize to them. You ask them to forgive you. Some can feel sorry for you while others won’t and they will tell you to fetch any other man.” (Client, <4 ANC visits, High-quality dispensary)

Partner presence was reported to be a barrier to accessing ANC services. Many clients had the experience of being turned away or reprimanded when attending clinic without a partner. As a result, some clients delayed seeking ANC services until late in pregnancy when a partner could accompany them. The lack of partner presence was also a barrier to facility-based delivery services. In these cases, those women who had not attended ANC were sometimes also denied a facility-based delivery.

“I went alone because my husband resisted. If you don’t go to the clinic and you don’t have the clinic card they reject you unless you have a letter from the village chairman. Then they prepare a card for you and take you to the delivery room. Without the letter, they won’t accept you. Maybe you can go to the traditional birth attendants.” (Client, <4 ANC visits, High-quality dispensary)

Service providers recognized that while it was important for clients to attend with their partners, clients should not be denied services if the partner was absent. In addition, providers remarked that clients who are turned away often do not return to the facility. As a result, these clients also fail to receive critical interventions for maternal and child health.

“Providers attend pregnant women who went and those who have not gone with their partners. This is still in education/mobilization though response is still low. In other places a woman is not attended at all if she has not gone with her partner. But for us, if she has not come with her partner, we attend her but we educate them on the importance of coming with the partner next visit.” (Provider, High-quality health center)

Some providers mentioned that it was a requirement to attend ANC with a partner. Providers insisted they would still provide services to those in critical need. However, some providers turned away non-critical patients if they did not attend with a partner.

“We serve both those who come with their husbands and those who do not come with husbands. As long as we get clear reasons for not coming with their partners since nowadays it is required to come with your partner. [If they provide good explanations] we give them services and if she fails to express herself we tell her to bring letter from the Village Executive Officer [VEO] otherwise the husband must come. If she is in critical condition you will give her services. So we look at the condition of the client, if she is not that serious we tell her the real situation.” (Provider, Low-quality dispensary)

## DISCUSSION

This study explored the quality of ANC services as experienced by Tanzanian clients and providers. Both clients and providers perceived quality of care as being comprised of items related to experience of care, provision of care, and cross-cutting essential physical and human resources. The results of our free-listing also showed that the experience of care was just as important to clients and providers as the availability of physical and human resources and the content of care delivered. These findings complement others from the literature on quality of care in this setting. Results from other studies in Tanzania similarly indicate that client’s perception of the quality of maternal care received is influenced by having a respectful, knowledgeable provider, availability of medicines, availability of medical equipment, and timely provision of services [27-32], and that the greatest predictor of health facility preference was kind treatment by the provider [33]. While past research establishes experience of care as an important component of a quality health service, the existing literature does not always clearly define what aspects are most important to clients and providers nor identify areas of overlapping importance between clients and providers. Our study shows that according to both clients and providers, a positive care experience should include good communication, listening to the client, treating clients respectfully, keeping patient confidentiality, attending to clients in a timely manner, and allocating sufficient time to each client.

This study also reveals the critical, and often underestimated, importance of the relationship between the patient care experience and the use of maternal health services [34]. Clients and providers emphasized that the continuation of ANC services and use of facility-based delivery services is influenced by good communication, politeness, and confidentiality during initial service provision. In addition, the experience of care can be a barrier to service utilization. Clients recounted how women of low socioeconomic status face discrimination and even denial of services if they cannot purchase required supplies at the time of delivery. Clients also reported that a partner presence requirement delayed and deterred ANC care-seeking, and prevented some women from utilizing facility-based delivery services. These barriers to care are especially relevant in Tanzania as coverage of one ANC visit is almost universal (98%), while coverage of four more ANC visits (51%) and skilled birth attendance at delivery (64%) remain persistently low [35]. This break in continuity of care from pregnancy to delivery slows progress in maternal mortality reduction in Tanzania. However, improvements in maternal health service utilization can be made by addressing these key facilitators and barriers to care.

Our findings also show that both clients and providers associate a positive patient experience with improved health outcomes for the mother and baby. This perception may encourage service utilization if other barriers are removed. ANC services are often thought of as a package of clinical interventions delivered to pregnant women. However, equally important is the communication of information to prepare women for birth as well as listening to women to detect potential pregnancy complications, reducing the risk of miscarriage, maternal mortality, and neonatal mortality [36]. Tanzania is one of ten countries that contributes to over 60% of maternal deaths worldwide. Therefore, this association between a positive patient experience and improved health outcomes is particularly relevant to those who develop policies to improve maternal health outcomes [35].

Finally, our findings show that human resource shortages are a limitation for providers striving to deliver high-quality services. Providers highlighted that the shortage of human resources resulted in long wait times and shorter duration of visits with clients. In Tanzania, there are only 4.4 physicians, nurses, and midwives per 10,000 population [37]. This falls far short of the recommended 23 physicians, nurses, and midwives per 10,000 population, the minimum level of health workers estimated to be required to achieve adequate coverage rates for primary health care interventions [38,39]. In addition, a study in Tanzania found that health facilities employed only 14% of the recommended number of nurses and 20% of the recommended number of clinical staff. Furthermore, 44% of the expected clinical staff were not present to deliver services, highlighting the severe shortage of human resources contributing to the health crisis in Tanzania [40]. Improving the human resource for health crisis may be an essential step in reducing barriers to delivery of high-quality maternal health services.

This study occurred at a critical juncture in policy-making in Tanzania, where the government has embarked on a new strategic plan to improve reproductive, maternal, newborn, child, and adolescent health (RMNCAH). A key focus of this plan is to improve the quality of services as this has been identified as a major challenge to reducing maternal and newborn mortality [41]. The findings from this study may help the government to incorporate components of experience of care within existing RMNCAH guidelines to improve service quality at health facilities and to develop metrics for assessing experience of care as part of national monitoring and evaluation of the quality of health services.

The findings from this study may also help inform the development of indicators and metrics for measuring experience of care globally. Our results highlight the importance of experience of care and identify good communication, listening to the client, treating clients respectfully, maintaining patient confidentiality, attending to clients in a timely manner, and allocating sufficient time to each client as components of experience of care that are valued by patients and providers should be considered for inclusion in quality of care measures. These findings align well with and reinforce the importance of global initiatives and frameworks on respectful maternity care [42]. However, more research is needed to define the best methodology for measuring experience of care.

This study has both limitations and strengths. First, we focused on a sample of clients and providers from only one region in Tanzania. However, we captured a range of perspectives: providers at differing facility types and levels of quality, and clients with diverse ANC experiences and utilization levels. The consistency of findings despite the heterogeneity of our sample suggests the findings could be transferable to other similar contexts. Second, the study did not include women who never used ANC services. Women who do not use ANC services are likely to have different perspectives and backgrounds from those who have attended even one ANC visit, but they also would not be able to comment on their personal experience of care. That said, since use of ANC for at least one visit is almost universal in Tanzania, our sample reflects the experience of the majority of pregnant women in Tanzania [35]. Finally, the sampling strategy classified facilities using an ANC quality of care score that focused on facility inputs and human resources. We chose this metric due to the availability of well-defined readiness indicators which utilize these items [43]. Although we also had data on patient experience from the TSPA, this data showed very low variability, and was not useful for differentiating facilities. We recognize that this is not a comprehensive quality metric. However, research has found facility inputs and human resources to be necessary, but not sufficient for quality services, so we feel this was a reasonable proxy [44,45].

## CONCLUSION

The experience of care in LMICs is an overlooked, yet critically important topic. Key to measuring and improving the quality of antenatal care is understanding the experience of care from those who receive and deliver services. Notably, measurement of quality of care has historically focused on facility inputs and provision of care. We highlight the importance of incorporating experience of care into future quality improvement activities and quality measures. By doing so, we identify barriers and facilitating factors of practical use to policy-makers and governments in LMICs who aim to improve ANC service coverage and reduce maternal mortality.
